# Draft genome sequence of *Rhodotorula* sp. BUB8, an oleaginous yeast isolated from Mt. Makiling Forest Reserve, Laguna, Philippines

**DOI:** 10.1128/mra.00377-24

**Published:** 2024-08-30

**Authors:** Irene G. Pajares, Kristine Rose M. Ramos, Andrew D. Montecillo, Francisco B. Elegado, Asuncion K. Raymundo

**Affiliations:** 1 National Institute of Molecular Biology and Biotechnology, University of the Philippines Los Baños, Los Baños, Laguna, Philippines; 2 Institute of Biological Sciences, University of the Philippines Los Baños, Los Baños, Laguna, Philippines; University of California Riverside, Riverside, California, USA

**Keywords:** oleaginous yeast, biofuel, biodiesel

## Abstract

This paper reports the draft genome sequence of *Rhodotorula* sp. BUB8, an oleaginous yeast isolated from the forest canopy of Mt. Makiling Forest Reserve in the Philippines. The draft genome is 20,394,133 bp with 64.3% guanine-cytosine (GC) content, 6,604 coding genes, 109 tRNA coding, and 6 snRNA-coding genes.

## ANNOUNCEMENT

Oleaginous yeasts are being explored for their potential to provide unique platforms for the sustainable production of fatty acid-derived oleochemicals due to their capability to synthesize and accumulate lipids mainly as triacylglycerides (1). *Rhodotorula* sp. BUB8 can produce significant amounts of microbial lipids from glucose and glycerol and share an almost similar fatty acid profile to vegetable oils (1).

*Rhodotorula* sp. BUB8 was isolated from the upper bark of a Bagtikan tree (*Parashorea malaanonan*) found in the Mt. Makiling Forest Reserve (2). The strain was cultured on yeast extract-peptone-dextrose (YEPD) media for 24 h at 30°C and was deposited to the Philippine Culture Collection of Microorganisms at the National Institute of Molecular Biology and Biotechnology, University of the Philippines Los Baños, with accession number 2345. Using the Basic Local Alignment Search Tool of the National Center for Biotechnology Information database, the ITS-5.8s rRNA gene (GenBank accession number OP808033.1) of BUB8 displayed 99% identity with *Rhodotorula paludigena* (2).

The genomic DNA of BUB8 was extracted from a 10-mL YEPD overnight culture using Quick DNA Fungal/Bacterial DNA Extraction Kit according to the manufacturer’s instructions (Zymo Research, Irvine, CA). Sequencing was conducted by Novogene Co., Ltd. (China), with the DNA library prepared using a Novogene NGS DNA library prep set (catalog no. Pt004), in which the DNA was randomly sheared into short fragments, end-repaired, A-tailed, then ligated with an Illumina adapter. The fragments with adapters were PCR amplified, size selected, and purified with solid-phase reversible immobilization beads (SPRI) beads. The library was checked with Qubit and quantified by real-time PCR. Fragment size distribution was determined using a bioanalyzer. Quantified libraries were pooled and sequenced on an Illumina MiSeq (Novogene Co., Ltd.). Reads were assessed using FastQC, and reads with low-quality nucleotides (*Q* value ≤38) exceeding 40 bp, containing N nucleotides exceeding 10 bp, and whose overlap with the adapter exceeded 15 bp by default were eliminated. The assembly was performed using SOAPdenovo (v.2.04) (3–7). Genome size was estimated by K-mer analysis using K-mer Analysis Toolkit (8), and the assembly quality was assessed using QUAST (Galaxy v.5.0.2 + Galaxy 5) (9). Contigs with less than 500 bp were removed. Default parameters were used with all tools unless otherwise specified.

**Fig 1:**
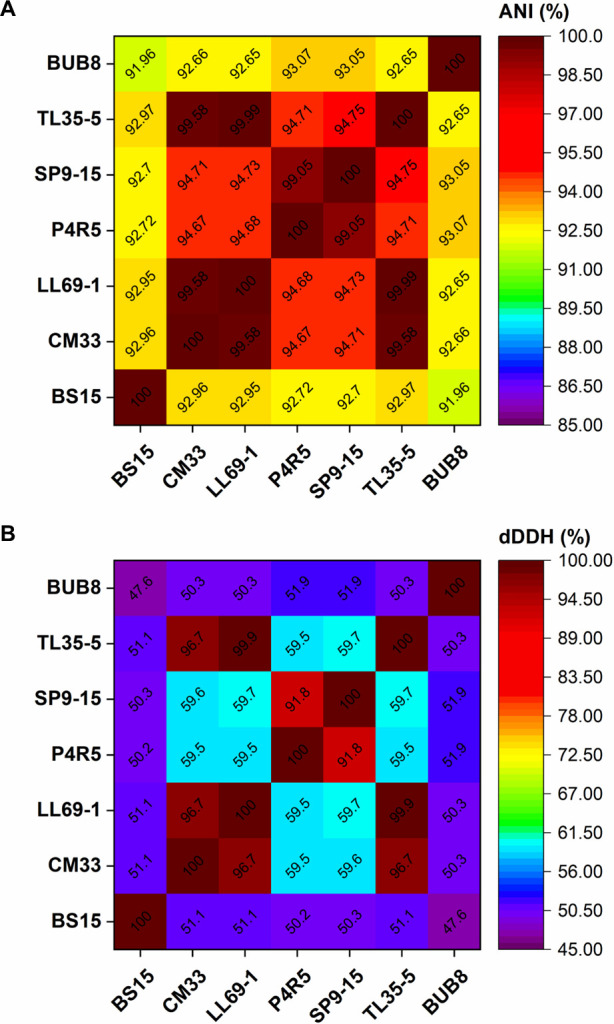
Comparative genomics average nucleotide identity (ANI) (**A**) and digital DNA-DNA hybridization (dDDH) (**B**) analyses of BUB8 with related *R. paludigena* strains. Genome accession numbers for *R. paludigena* strains: BS15 (GCA_036244875.1), CM33 (GCA_005281665.1), LL69-1 (GCA_033458335.1), P4R5 (GCA_019671115.1), SP9-15 (GCA_029873615.1), and TL35-5 (GCA_028828615.1).

A total of 13,788,111 and 12,998,269 of raw and filtered paired-end reads were generated, respectively. The reads assembled into 54 scaffolds with a total length of 20,394,133 bp, N50 of 536,656 bp, GC content of 64.3%, and an estimated mean sequencing depth of 162×. The nearest neighbor identity of BUB8 genome based on genome-relatedness indices (i.e., average nucleotide identity and digital DNA-DNA hybridization) was identified using the Enveomics Average Nucleotide Identity Calculator (http://enve-omics.ce.gatech.edu/ani/) and ANI-independent Genome-to-Genome Distance Calculator (v.3.0) online tools (https://ggdc.dsmz.de/ggdc.php#) (10, 11). BUB8 was most closely related to P4R5 (GCA_019671115.1) with 93.07% and 51.9% identities, respectively, among *R. paludigena* strains (12–15). These values are below the generally accepted cut-off for species boundary of 95% and 70%, suggesting BUB8 is a novel species of *Rhodotorula* (Fig. 1).

## Data Availability

The draft genome sequence of *Rhodotorula* sp. BUB8 has been deposited in GenBank under accession number JAZHGP000000000, BioProject number PRJNA1051797, and Sequence Read Archive accession number SRX24721207.
